# Stress and polycystic ovarian morphology in functional hypothalamic amenorrhea: a retrospective cohort study

**DOI:** 10.1186/s12958-023-01095-5

**Published:** 2023-05-11

**Authors:** Marlene Hager, Didier Dewailly, Rodrig Marculescu, Stefan Ghobrial, John Preston Parry, Johannes Ott

**Affiliations:** 1 Clinical Division of Gynecological Endocrinology and Reproductive Medicine, Department of Obstetrics and Gynecology, Medical University of Vienna, Vienna, Austria; 2 Faculty of Medicine Henri Warembourg, University of Lille, Lille Cedex, France; 3 Department of Laboratory Medicine, Medical University of Vienna, Vienna, Austria; 4 Department of Obstetrics and Gynecology, Parryscope and Positive Steps Fertility, Louisiana State University Health, Madison, Shreveport, Mississippi, Louisiana USA; 5 Department of Obstetrics and Gynecology, University of Mississippi Medical Center, Jackson, MS USA

**Keywords:** Hypogonadotropic hypogonadism, Stress, Polycystic ovary syndrome

## Abstract

**Background:**

Women with functional hypothalamic amenorrhea (FHA) reveal polycystic ovarian morphology (PCOM) in up to 50%. If stress sensitivity in women with polycystic ovary syndrome (PCOS) is the reason why PCOS women are prone to develop FHA, patients with FHA caused by stress should reveal PCOM more often.

**Methods:**

In a retrospective cohort study, 38 stress-associated and 38 excessive exercise-induced FHA women were included. The main outcome parameter was PCOM. In addition, the focus was on general patient characteristics as well as on prolactin, dehydroepiandrosterone-sulphate (DHEAS), and anti-Mullerian hormone (AMH).

**Results:**

PCOM was found in 34/76 patients (44.7%). The stress group showed a higher prevalence of PCOM than the excessive exercise group (57.9% versus 31.6%, *p =* 0.019) as well as higher prolactin levels (median 13.2ng/mL versus 11.7ng/mL, *p =* 0.008) and a trend towards higher DHEAS levels (*p =* 0.058).

**Conclusions:**

In FHA women, the PCOM prevalence was significantly higher in the stress-group than in the excessive exercise-group. The well-known stress sensitivity in women with PCOS might explain why PCOS women are prone to develop FHA as well as the high PCOM prevalence in FHA women.

## Background

Women with functional hypothalamic amenorrhea (FHA) can overlap with polycystic ovarian morphology (PCOM) as frequently as 50% of cases. This prevalence for PCOM in women with FHA is higher than the one observed for the general population (range: 7-24%) [1]. The exact reasons remain unclear, why FHA women could be more prone to develop PCOM, and whether women with polycystic ovary syndrome (PCOS) are at increased risk for developing FHA. Regardless, women with FHA-PCOM demonstrate features that are similar to those observed in PCOS, such as high serum anti-Mullerian hormone (AMH), hyper-responsiveness of luteinizing hormone (LH) to a Gonadotropin releasing hormone (GnRH) bolus and insulin resistance [1–3]. These similarities have led some to suggest that some FHA-PCOM patients initially had simple PCOS [1–3].

Women with PCOS seem to have a generalized increase in sympathetic nerve activity, suggesting that they suffer from increased stress and/or less adequate response to stressors [4]. Stress and stress sensitivity are well-recognized causes for FHA [5]. If this stress sensitivity in PCOS is the reason why PCOS women are prone to develop FHA, patients with FHA caused by stress alone should reveal PCOM more often than those who developed FHA based on excessive exercise. In addition, there may even be behavioral biases driving the overlap, where women with hyperandrogenism may have hormonally driven excessive exercise habits or women with PCOS exercise to avoid androgen associated weight gain. We aimed to evaluate this in a retrospective cohort study.

## Methods

### Study population and study design

This retrospective cohort study was conducted at the Clinical Division of Gynecologic Endocrinology and Reproductive Medicine of the Medical University of Vienna, Austria. We estimated that the prevalence of PCOM would be 40% in our FHA population [2] and that stress would increase the prevalence of PCOM (55% versus 25%). With these estimates, a sample size of 38 patients per group would be necessary to detect a difference in stress-associated FHA-PCOM relative to exercise-associated FHA (power 95%, *alpha =* 0.05). Thus, 76 FHA women (38 “stress-associated”, 38 “excessive exercise”) were included from January 2017 – March 2022. As previously described [2, 3, 6], a strict FHA definition was used (also see Table 1): secondary amenorrhea for at least six months and a negative progestogen challenge test with context of intense physical activity or notion of recent psychological stress, confirmed by a psychologic report. Pregnancy, hypothyroidism, and hyperprolactinemia and any organ-related pituitary dysfunction had to be excluded. Moreover, a BMI > 30.0 kg/m^2^ was also an exclusion criterion to exclude women with obesity-related FHA. The study was approved by the local ethics committee (IRB number 1019/2023).

**Table 1:** Inclusion and exclusion criteria

Inclusion criteria		Exclusion criteria
Secondary amenorrhea for ≥ 6 months		Pregnancy
Negative progestin test with oral dydrogesterone 10 mg twice a day for ten days		Hypothyroidism
Together with at least one of the following:		Acne, hirsutism
	- *Excessive exercise group*: Exercising at least 10 h per week, which included any type of exercise (dancing, aerobics, biking, etc.) or running at least 30 miles per week	Psychiatric diseases using DSM IV criteria
	- *Stress group*: History of emotionally stressful events preceding the onset of amenorrhea included problems within the family, at school, at work or of psychosocial stress	Eating disorders according to the ICD-10 criteria
		BMI < 18.5 kg/m^2^ and BMI > 30.0 kg/m^2^

### Parameters analyzed

The AKIM-software was used for data acquisition. Blood samples were obtained during amenorrhea and were analyzed at the local ISO-certified Department of Laboratory Medicine, General Hospital of Vienna, Vienna, Austria according to ISO 15,189 quality standards: estradiol, follicle-stimulating hormone (FSH), LH, prolactin, AMH, testosterone, dehydroepiandrosterone-sulphate (DHEAS), and sex hormone-binding globulin (SHBG) were measured by the corresponding Cobas electrochemiluminescence immunoassays (ECLIA) on Cobas e 602 analyzers (Roche, Mannheim, Germany). On the same day, a vaginal ultrasound was performed with an “Aloka Prosound 6” ultrasound machine and an “UST-9124 Intra Cavity transducer” (frequency range 3.0–7.5 MHz; Wiener Neudorf, Austria). PCOM, the main outcome parameter, was defined as when the number of follicles per ovary exceeded 12 [2, 3]. In addition, the following basic patient characteristics were also included: age at evaluation, body mass index (BMI), and the duration of amenorrhea.

### Statistical analysis

Continuous variables are presented as medians with interquartile ranges (IQR), categorical parameters as numbers and frequencies. Factors associated with presence of PCOM were tested using a multivariable binary logistic regression model. For this model, odds ratios (OR) with their 95% confidence intervals (95%CI) and p-values are provided. Using the IBM Statistical Package for Social Science software 25.0 (level of significance: *p <* 0.05), groups were compared with the analysis of variance (ANOVA) and the Fisher’s exact test.

## Results

PCOM was found in 34/76 patients (44.7%). The stress group showed a higher prevalence of PCOM than the excessive exercise group (57.9% versus 31.6%, *p =* 0.019). A longer previous duration of amenorrhea in the excessive exercise group (*p =* 0.030) was noted (Table 2). However, BMI, testosterone, and SHBG levels were similar between the groups (Table 2). Of interest, not only was PCOM increased in stress-associated FHA relative to exercise-associated (57.9% versus 31.6%, *p =* 0.019), but prolactin was also significantly higher in patients with stress-associated FHA (median 13.2ng/mL, IQR 10.5–18.7 versus median 11.7ng/mL, IQR 9.3–14.4; *p =* 0.008). In addition, adrenal androgens (DHEAS) were also increased 1.32 fold (median 2.43 µg/mL, IQR 1.50–3.21 versus median 1.84 µg/mL, IQR 1.31–2.61; *p =* 0.058). Also of note, AMH was 1.56 fold higher in patients with stress-associated FHA than in exercise-associated FHA (median 3.87 ng/mL, IQR 2.23–6.22 versus median 2.48ng/mL, IQR 1.68–4.82), though as with adrenal androgens, this finding was not statistically significant (*p =* 0.153).

**Table 2:** Main results according to FHA cause

	Stress-induced FHA	FHA due to excessive exercise	p
Age (years) *	25.5 (21.0;30.0)	26.0 (22.8;29.0)	0.962
BMI (kg/m^2^) *	20.0 (18.8;22.8)	19.7 (18.7;21.8)	0.467
Duration of amenorrhea (months) *	12.0 (5.8;24.0)	17.0 (11.8;36.0)	0.030
Prolactin (ng/mL)	13.2 (10.5;18.7)	11.7 (9.3; 14.4)	0.008
Follicle stimulating hormone (mIU/mL) *	4.6 (3.2;6.1)	3.7 (2.1;5.5)	0.333
Luteinizing hormone (mIU/mL) *	2.5 (1.0;4.5)	1.6 (0.7;3.0)	0.298
Estradiol (pg/mL) *	23.5 (13.3;36.3)	19.5 (11.2;27.3)	0.168
Testosterone (ng/mL) *	0.22 (0.14;0.32)	0.20 (0.13;0.30)	0.619
Dehydroepiandrosterone-sulphate (µg/mL) *	2.43 (1.50;3.21)	1.84 (1.31;2.61)	0.058
Sexual hormone binding globulin (nmol/L) *	68.7 (55.1;101.1)	73.4 (56.3;102.8)	0.407
AMH (ng/mL) *	3.87 (2.23;6.22)	2.48 (1.68;4.82)	0.153
PCOM ^#^	22 (57.9)	12 (31.6)	0.019

Data are presented as *median (IQR) or ^#^ n (%)

A binary logistic regression model was used to evaluate whether age, BMI, the duration of amenorrhea and the cause for FHA were associated with the presence of PCOM were tested (Table 3). Notably, only women with stress-induced FHA revealed a higher risk for PCOM (OR 3.001, 95%CI: 1.134–7.964; *p =* 0.027).

**Table 3:** Binary logistic regression model: factors associated with presence of PCOM

	PCOM	Non-PCOM	OR (95%CI)	p
Age (years)*	25.0 (22.0;29.3)	26.0 (22.0;28.3)	0.989 (0.896;1.093)	0.832
BMI (kg/m^2^)*	19.7 (18.7;22.8)	19.9 (18.8;21.7)	1.018 (0.810;1.279)	0.878
Duration of amenorrhea (months)*	13.5 (9.5;24.0)	15.0 (6.8;24.0)	1.002 (0.973;1.031)	0.916
Stress-induced FHA (versus FHA due to excessive exercise)^#^	13.2 (10.5;18.7)	11.7 (9.3; 14.4)	3.001 (1.134;7.964)	0.027

Data are presented as *median (IQR) or ^#^ n (%)

## Discussion

Our findings were consistent with previous reports [1–3, 7], where a high overall prevalence of PCOM in FHA patients was found (about 45%). As hypothesized, the stress-associated FHA group revealed a significantly higher prevalence of PCOM compared to the excessive exercise group (Table 2). Stress versus excessive exercise was the most important parameter associated with PCOM (Table 3). Women with stress-induced had higher mean AMH levels, as previously reported in women with FHA and PCOM compared to those without PCOM [1–3]. Though these differences were not statistically significant, there were only ten more patients with PCOM in the stress group, which may mean that the lack of a difference was attributable to sample size. Keeping in mind that women with PCOS reveal a generalized increase in sympathetic nerve activity, which suggests that they suffer from increased stress and/or less adequate response to stressors [4], and that stress and stress sensitivity are well-recognized causes for FHA [5], our data support a link between stress and the presence of PCOM.

Notably, literature about the influence of stress on AMH levels is scarce. In non-PCOS women seeking infertility treatment, high stress levels did not correlate with AMH levels [8]. Cortisol is a well-known stress-biomarker [9]. While DHEA has been reported to be associated with cortisol levels, very low DHEA also negatively affected total testosterone [10]. It is well-established that abnormally low testosterone inhibits growth of small growing follicles, which then leads to reductions in granulosa cell mass and AMH levels [11]. However, this might not be true for women with PCOS, where high AMH is a marker of disease severity and likely the main disruptor of normal ovarian function [12] and usually higher cortisol levels are found than in controls [13]. In fact, we will not be sure about the changes which happen in women who develop FHA until we will be able to acquire longitudinal data.

In addition to the rate of PCOM, there were higher prolactin as well as a trend towards higher DHEAS levels in stress-induced FHA (*p =* 0.058). Chronic stress induces an intense cortisol production via an increased ACTH production and release from the POMC-neurons. At the same time, these neurons also secrete GABA, which acts as a stimulator of prolactin and can therefore lead to increased prolactin secretion [14]. Notably, prolactin has been mentioned as a possible serum biomarker of chronic stress and was found to be elevated in patients with burn out [15]. In addition, there was a trend towards higher DHEAS levels in stress-associated FHA. Due to stress-induced adrenal activity, this may be why adrenal androgens may be increased to a greater extent than ovarian androgens. Notably, DHEAS has also been claimed to be linked to chronic stress [15].

However, an alternative explanation could include underlying PCOS predisposing patients to stress, as PCOS is associated with higher rates of mood disorders, including depression.

The study limitations, which need to be addressed, include the fact that matching of age and of duration of amenorrhea was not possible and the retrospective study design, which might have introduced some kind of selection bias despite the strict in- and exclusion criteria (Table 1). Moreover, we cannot provide the exposure time to either excessive exercise or stress. This should also be seen as a limitation, since, hypothetically, exposure time might be associated with the development of PCOM, despite the fact that the duration of amenorrhea was not of relevance (Table 3). One could also argue that the sample size was comparably small. However, only patients with FHA diagnosed by strict criteria were included. In addition, FHA patients with either stress or excessive exercise only - which excludes patients with a combination of both, weight loss, and/or eating disorders - are rare to find.

## Conclusions

Women with stress-associated FHA revealed a higher PCOM prevalence than patients with FHA associated with excessive exercise. The well-known stress sensitivity in women with PCOS might explain why PCOS women are prone to develop FHA, which then could give an explanation for the high PCOM prevalence in FHA women. Although the presented results seem reasonable, they can only provide a background for the mentioned hypothesis. Longitudinal data of different stages of FHA will be needed for a definite statement.

## Data Availability

The datasets used and/or analysed during the current study are available from the corresponding author on reasonable request.

## List of Abbreviations

ANOVA: Analysis of variance
AMH: Anti-Müllerian hormone
BMI: Body mass index
DHEA-S: Dehydroepiandrosterone-sulphate
FHA: Functional hypothalamic amenorrhea
FSH: Follicle stimulating hormone
IQR: Interquartile range
LH: Luteinizing hormone
OR: Odds ratio
PCOM: Polycystic ovarian morphology
SHBG: Sexual hormone binding globulin
95%CI: 95% confidence interval
